# Significant interplay of BMI and Anthropometric Profile on Gender-Specific Health Indicators

**DOI:** 10.12669/pjms.41.5.11780

**Published:** 2025-05

**Authors:** Ayesha Sadiqa, Amna Nadeem, Aiza Rehman

**Affiliations:** 1 Ayesha Sadiqa, Physiology Department-IOD at CMH Lahore, Medical College and Institute of Dentistry, Lahore, Punjab, Pakistan; 2 Amna Nadeem, Department of Physiology, IMBB, The University of Lahore, Lahore, Pakistan; 3 Aiza Rehman, Physiology Department-IOD at CMH Lahore, Medical College and Institute of Dentistry, Lahore, Punjab, Pakistan

**Keywords:** Anthropometric Measurements, Blood pressure, Body Mass Index, Fasting Blood Sugar, Pulse rate, Sex Differences

## Abstract

**Objectives:**

To investigate gender-based relationships among BMI, arm/chest circumference, and their influence on heart rate (HR), BP, and fasting blood sugar (FBS).

**Methods:**

A descriptive-analytical study was conducted with 157 participants aged 18-28 at the University of Lahore after institutional ethical approval (REG/GRT/22/AHS-129) from December 2022 to April 2023. Arm/chest circumference was measured. BMI was calculated. Standard protocols were followed to measure BP and HR. FBS was assessed through biochemical testing. A t-test and ANOVA were used to compare variables. For associations, the Pearson correlation was used.

**Results:**

Significant differences were noted among all categories of BMI, HR, systolic-BP, and FBS. Females had a BMI 14.9% lower than males in overweight/obese (p=0.021). In the normal-HR category, females showed rates 5.82% lower than males (p=0.0002), while in the normal-BP category, females comprised 83.83% compared to 16.16% for males (p=0.027). Males had an FBS 3.79% higher than females in elevated-FBS (p=0.039) despite 70% of those affected being female. A positive correlation between FBS and HR was found in borderline and normal FBS. Being overweight/obese was positively associated with systolic-BP (p=0.006), diastolic-BP (p=0.011), arm-circumference (p=0.040), and chest circumference (p=0.018).

**Conclusion:**

Overweight/obese females had a lower BMI but higher FBS than males, who had higher HR and BP. FBS is positively associated with HR in borderline and normal FBS categories. BMI significantly influenced arm/chest size, BP, and FBS. All overweight/obese females expressed a positive association with BP as well as arm and a negative association with HR.

## INTRODUCTION

Heart rate (HR), blood pressure (BP), and fasting blood sugar (FBS) are the key physiological indicators that reflect cardiovascular and metabolic health. Elevated HR, BP, and FBS are associated with an increased risk of cardiovascular diseases, diabetes, and other chronic conditions.^1^ Understanding the factors influencing these parameters is essential for early disease detection. Anthropometries like as body mass index (BMI), arm-circumference, and chest-circumference, are crucial indicators of overall health and disease risk.^1^,^2^

The literature suggests that gender can impact human cardiovascular and metabolic health because of varied anthropometries.^3^ By understanding these gender-specific differences, tailored interventions can be developed to address the unique needs of different populations. These measurements provide valuable insights into fat distribution and metabolic health.^2^,^3^ However, the interplay between these factors and cardiovascular health, particularly about gender differences, remains incompletely understood.

BMI, a widely used indicator of body fat derived from weight and height, is closely linked to health outcomes like HR, BP, and FBS.^4^ However, the complex relationship between BMI, gender-specific anthropometric profiles, and physiological markers such as HR, BP, and FBS remains underexplored.^4^,^5^ Obesity, as indicated by a high BMI, poses a significant global health challenge, increasing the risk of hypertension, diabetes, and cardiovascular diseases.^6^ Excess body fat has been shown to elevate cardiac workload and trigger systemic inflammation, linking obesity to changes in HR and BP.^5^,^6^ Additionally, anthropometries have emerged as complementary indicators of body composition and cardiovascular risk, particularly across diverse BMI categories.^2^,^6^,^7^

Both genders in fat distribution, hormonal regulation, and physiological stress responses lead to varying health outcomes.^6^,^7^ Women generally have lower BP but are more susceptible to diabetes at a similar BMI as men.^8^,^9^ Differences in HR have been attributed to autonomic nervous system regulation, with women showing higher resting HR and more significant HR variability.^10^,^11^ Despite these established patterns, limited studies explore the combined influence of BMI and anthropometric profiles on physiological parameters within a gender-specific framework.

This research highlights the link between body measurements and physiological parameters, as a foundation for future studies and personalized health strategies. It aims to investigate gender-based relationships among BMI and anthropometries (arm/chest circumference) and their influence on HR, systolic BP, and FBS.

## METHODS

A descriptive-analytical study was conducted with nursing undergraduate students aged 18-28 years from December-2022 to April-2023 at the University of Lahore. A convenience sampling technique was employed. Out of 157 participants, 39 were males, and 118 were females. Informed consent was taken from all. Students with good physical health were included, and those with infections or who were on anticonvulsant therapy or other health issues were excluded.

### Ethical Approval:

Sampling was initiated after ethical approval, referenced by REG/GRT/22/AHS-129; Dated: November 8, 2022.

Participants’ heights were measured in meters using a wall-mounted measuring tape. They stand barefoot on the floor, with their heads and buttocks touching the wall. On a weighing scale, the body weight was measured with minimal clothing (kg). BMI was calculated using the standard metric of kg/m². Chest-circumference was measured (inches) through a measuring tape at the mesosternale landmark, warranting the perpendicular axis of the thorax. Participants were asked to remain relaxed with their arms separated for tape placement. Recording completed after exhale. Mid-upper arm Circumference (MUAC) was measured on the right arm (inches) via measuring tape following a standard protocol.

The ‘three-finger method’ was used to assess HR (beats/minute). Systolic/diastolic BP was measured (mm of Hg) through a mercury sphygmomanometer and a stethoscope. Participants were provided a 15-minute relaxation time before BP measurement. FBS was estimated (mg/ml) through biochemical blood sample analysis, where the sample was taken after 8-hours of fasting.

### Statistical analysis:

Statistical analysis was conducted via SPSS version 24.0. The proportional imbalance between genders was first statistically adjusted through the flexible application feature of the Bootstrapping technique in SPSS. Mean, Number, and Standard deviation expressed the descriptive statistics. A t-test and one-way ANOVA were applied to observe the statistical difference between the two groups and three groups, respectively.

## RESULTS

Significant differences were revealed across all three BMI categories: 56.01% had normal weight, 34.39% were underweight, and 9.55% were overweight/obese. A significant difference was found among HR categories, with 50.31% normal, 32.5% borderline, and 17.2% elevated. Systolic BP showed a significant difference between elevated and normal, where 63.05% had normal and 36.9% had elevated levels. FBS-categories differed significantly, with 63.69% normal, 32.5% elevated, and 3.2% reduced (Table-I).

**Table-I:** Statistical difference among categories of study variables.

Variables	Mean±S.D.	Categories	N=157	Mean±S.D.	P-value
BMI (kg/m^2^)	20.58±3.98	Overweight/obese(≥25)	15	29.39±3.84	0.0000
		Normal-weight(18.5-24.9)	88	21.22±1.89	
		Underweight(<18.5)	54	17.03±0.99	
HR (beats/min)	82.21±10.16	Elevated(>90)	27	98.07±7.36	<0.00001
		Borderline(>80-90)	51	85.96±2.66	
		Normal(60-80)	79	74.36±5.05	
Systolic-BP (mmHg)	110.45±9.88	Elevated(≥120)	58	120.68±2.55	<0.0001
		Normal(<120)	99	104.45±7.31	
FBS (mg/dl)	97.35±11.69	Elevated(>100)	51	110.61±4.82	0.0000
		Normal(70-100)	100	91.33±7.65	
		Reduced(<70)	5	69.6±0.89	

A significant difference in BMI categories was found between genders, with 73.3% of females being overweight/obese and 26.6% of males. Females had 14.9% lower BMI than males in this category. A non-significant gender-based difference was noted in normal-weight category, though females (71.26%) were still outnumbered. In underweights, females displayed a non-significant 2.24% lower BMI than males. No gender differences were expressed in elevated and borderline HR, although females had slightly higher HR in both categories. A significant difference in the normal HR category was observed, with females’ HR 5.82% higher than males’. No significant difference was found in systolic-BP in elevated category; however, in the normal category, females comprised 83.83% compared to males’ 16.16%, with females showing a 4.05% lower systolic-BP. In ‘elevated FBS-category’, males had 3.79% higher levels than females, but 70% of those affected were females. In ‘normal FBS-category’, no significant gender differences were observed. Mainly females were present in ‘reduced FBS-category’ while a single male falls in this category, making up 4.27% of females (Table-II).

**Table-II:** Gender-based statistical difference in each category of study variables.

Variable		Groups	N	Mean±S.D.	P-value
BMI	Overweight/obese(≥25)	Males	4	33.02±4.19	0.021
		Females	11	28.08±2.86	
	Normal-weight(18.5-24.9)	Males	25	21.23±1.86	0.964
		Females	62	21.21±1.91	
	Underweight(<18.5)	Males	10	17.34±0.70	0.265
		Females	44	16.95±1.04	
HR	Elevated(>90)	Males	5	97.00±2.75	0.774
		Females	22	98.09±8.17	
	Borderline(>80-90)	Males	12	85.16±1.19	0.235
		Females	39	86.21±2.94	
	Normal(60-80)	Males	22	77.63±3.69	0.0002
		Females	57	73.11±4.95	
Systolic-BP	Elevated(≥120)	Males	23	120.86±2.88	0.676
		Females	35	120.57±2.35	
	Normal(<120)	Males	16	108.12±4.03	0.027
		Females	83	103.74±7.59	
FBS	Elevated(>100)	Males	30	93.73±5.65	0.039
		Females	70	90.3±8.19	
	Normal(70-100)	Males	9	113.11±7.11	0.977
		Females	42	110.07±4.09	
	Reduced(<70)	Males	1	69.01±0.0	-
		Females	5	69.6±0.89	

BMI was negatively associated with elevated and borderline HR-categories but positively associated with normal HR-categories across all study groups. Systolic-BP showed a non-significant positive relation with elevated and borderline HR-categories in total participants and females, while a non-significant negative relation was found in males. However, males with normal-HR had a significant positive association. For diastolic-BP, a non-significant association with HR was noted in ‘elevated category’ for all groups and among males in borderline category; however, females showed a slight positive association. In normal HR-category, significant positive associations were found between diastolic-BP and HR for total participants and males, while females had a mild, non-significant association. A non-significant negative association existed between FBS and HR in total participants and females in the elevated category. Still, strong positive relations were noted in borderline and normal HR-categories. Arm-circumference showed a non-significant negative association with HR across categories for total participants and females but a significant negative relation for males in ‘elevated category’. Chest-circumference demonstrated mixed associations: non-significant positive for total participants and females in elevated/borderline categories, but moderate negative for males. In ‘normal HR-category’, negative associations were found among total participants and females, with significant results in females, while males were significantly related to HR (Table-III).

**Table-III:** Correlational of the study population for each category of HR & Systolic-BP.

Correlational-variables	Groups	HR-Categories								
		Elevated			Borderline			Normal		
		Pearson-r	P-value		Pearson-r		P-value	Pearson-r		P-value
BMI	Total	-0.203	0.309		-0.114		0.350	0.204		0.071
	Males	-0.294	0.631		-0.315		0.183	0.046		0.838
	Females	-0.207	0.355		-0.007		0.961	0.136		0.313
Systolic-BP	Total	0.046	0.817		0.055		0.650	0.176		0.120
	Males	-0.310	0.611		-0.262		0.264	0.604		0.0029
	Females	0.070	0.756		0.186		0.200	-0.151		0.262
Diastolic-BP	Total	-0.084	0.677		-0.104		0.395	0.230		0.041
	Males	-0.310	0.611		-0.382		0.098	0.726		0.0001
	Females	-0.086	0.703		0.034		0.811	-0.125		0.354
FBG	Total	-0.271	0.171		0.922		<0.00001	0.925		<0.00001
	Males	0.645	0.239		0.922		<0.00001	0.886		<0.00001
	Females	-0.316	0.151		0.934		<0.00001	0.942		<0.00001
Arm-circum.	Total	-0.068	0.736		-0.088		0.472	-0.009		0.937
	Males	-0.988	0.003		0.002		0.992	0.249		0.263
	Females	-0.051	0.821		-0.033		0. 821	-0.207		0.122
Chest-circum.	Total	0.017	0.932		0.105		0.390	-0.037		0.746
	Males	-0.652	0.235		-0.331		0.154	0.475		0.025
	Females	0.024	0.915		0.274		0.056	-0.332		0.0116
		*Systolic BP-Categories*								
	*Elevated*				*Normal*				
	*Pearson-r*		*P-value*		*Pearson-r*			*P-value*		
BMI	Total	-0.078		0.560		0.091			0.367	
	Males	-0.238		0.274		-0.056			0.836	
	Females	0.086		0.622		0.095			0.388	
Diastolic-BP	Total	0.038		0.773		0.778			<0.00001	
	Males	1		<0.00001		0.677			0.003	
	Females	-0.364		0.033		0.775			<0.00001	
HR	Total	0.168		0.205		0.227			0.023	
	Males	0.370		0.081		0.086			0.750	
	Females	0.127		0.465		0.234			0.032	
FBG	Total	-0.030		0.952		-0.053			0.602	
	Males	0.147		0.503		-0.426			0.099	
	Females	-0.134		0.442		-0.030			0.787	
Arm-circum.	Total	0.086		0.517		0.019			0.851	
	Males	0.086		0.696		-0.134			0.631	
	Females	0.072		0.681		0.005			0.959	
Chest-circum.	Total	0.068		0.610		-0.022			0.828	
	Males	-0.295		0.179		0.019			0.941	
	Females	0.163		0.0347		-0.057			0.608	

A non-significant negative correlation was found between BMI and systolic-BP in males, while females showed a non-significant positive association in elevated and normal systolic-BP categories. ‘Elevated systolic-BP’ had a mild negative correlation, while a mild positive correlation was observed in the ‘normal systolic-BP among all participants. No significant relationship between diastolic and systolic-BP was found in ‘elevated category’ for the total group. Still, significant gender-based associations were noted, with strong positive and mild negative correlations in males. Strong associations were present across all study groups in the normal systolic-BP group (Table-III).

A direct relationship between HR and systolic-BP was found in both systolic categories, with significant findings limited to total participants and females in ‘normal category’. An inverse relationship between FBG and BP was observed in all groups for both systolic categories, except males in the elevated category, who showed a non-significant direct association. Mild positive relationships between arm-circumference and systolic-BP were noted in both categories, except for males in the ‘normal category’, who displayed a negative association. A positive association between chest circumference and systolic BP was significant in females within the elevated systolic category, while males showed a mild inverse relationship. Conversely, females and total participants in ‘normal category’ exhibited a non-significant inverse relationship, with males showing a mild direct association (Table-III).

A non-significant negative association was observed between HR and BMI across all groups, including underweight and overweight/obese categories. A mild, non-significant positive relationship was noted for the normal-weight group, while a non-significant inverse association between HR and BMI was noticed in all three study groups. In terms of systolic-BP, a slight positive association with BMI was found in all categories, with significant results in the total and female category of overweight/obese BMI. For diastolic-BP, all categories showed similar trends, except underweight females, who had a negative relationship, while only the total overweight/obese group showed significant association. FBG displayed a positive but non-significant association with BMI for females and total participants. A non-significant negative association between FBG and BMI was found among males in all three BMI categories. Overall, a positive association was found between arm/chest size and BMI of each category, but a significant positive association was noticed only for overweight/obese BMI category (Table-IV).

**Table-IV:** Correlational of the study population for each category of BMI & FBS.

Correlational-variables	Groups	BMI-Categories					
		Underweight		Normal-weight		Overweight/obese	
		Pearson-r	P-value	Pearson-r	P-value	Pearson-r	P-value
HR	Total	-0.208	0.131	0.022	0.839	-0.433	0.106
	Males	-0.375	0.285	0.006	0.974	-0.558	0.441
	Females	-0.159	0.302	0.027	0.830	-0.365	0.276
Systolic-BP	Total	0.111	0.421	0.164	0.128	0.665	0.006
	Males	0.379	0.279	0.064	0.759	0.643	0.356
	Females	0.017	0.911	0.197	0.123	0.605	0.048
Diastolic-BP	Total	0.032	0.818	0.158	0.142	0.631	0.011
	Males	0.379	0.279	0.138	0.508	0.643	0.356
	Females	-0.087	0.574	0.172	0.181	0.557	0.074
FBG	Total	0.158	0.252	0.001	0.990	0.079	0.778
	Males	-0.111	0.762	-0.006	0.977	-0.564	0.435
	Females	0.186	0.224	0.003	0.982	0.328	0.324
Arm-circum.	Total	0.200	0.146	0.035	0.745	0.533	0.040
	Males	0.257	0.473	0.124	0.554	0.405	0.594
	Females	0.157	0.307	0.011	0.935	0.351	0.288
Chest-circum.	Total	0.225	0.101	0.111	0.302	0.598	0.018
	Males	0.298	0.402	0.279	0.176	0.802	0.197
	Females	0.195	0.202	0.092	0.476	0.310	0.352
	*FBS-Categories*						
	*Elevated*		*Normal*		*Reduced*	
	*Pearson-r*	*P-value*	*Pearson-r*	*P-value*	*Pearson-r*	*P-value*
BMI	Total	0.201	0.103	0.163	0.103	0.660	0.052
	Males	0.144	0.527	-0.120	0.527	0.126	0.873
	Females	0.276	0.046	0.238	0.046	-0.859	0.062
Systolic-BP	Total	0.082	0.037	0.208	0.037	-0.217	0.574
	Males	0.155	0.0685	0.337	0.0685	0.126	0.873
	Females	0.048	0.436	0.094	0.436	-0.225	0.715
Diastolic-BP	Total	0.101	0.037	0.208	0.037	-0.217	0.574
	Males	0.155	0.011	0.456	0.011	0.126	0.873
	Females	0.069	0.648	0.055	0.648	-0.225	0.715
HR	Total	0.917	<0.00001	0.941	<0.00001	0.594	0.091
	Males	0.959	<0.00001	0.938	<0.00001	0.126	0.873
	Females	0.89	<0.00001	0.949	<0.00001	0.592	0.292
Arm-circum.	Total	0.130	0.034	-0.212	0.034	-0.318	0.403
	Males	-0.059	0.219	-0.231	0.219	-0.239	0.760
	Females	0.178	0.019	-0.279	0.019	0.172	0.782
Chest-circum.	Total	-0.305	0.411	-0.083	0.411	-0.309	0.418
	Males	-0.067	0.286	0.201	0.286	0.345	0.654
	Females	-0.461	0.067	-0.222	0.067	0.408	0.495

All groups found a non-significant direct association between BMI and elevated FBS, but only females showed a statistically significant link. Similarly, a significant association was observed between BMI and FBS in the normal category for females, while males exhibited a non-significant negative relationship. Total participant group and female group with reduced-FBS, showing a non-significant negative relationship with systolic-BP. A direct association between systolic BP and FBS was noted across all groups for both elevated and normal FBS categories, with significant links in total participants. Diastolic-BP also had significant positive associations in males and total participants who were with normal and elevated FBS. HR was significantly associated with FBS in elevated and normal categories across all groups; however, all groups with the reduced-FBS showed a non-significant link. Arm-circumference positively and significantly correlated with elevated-FBS for total participants and females, while males in the same category had a non-significant inverse relationship. Arm-circumference showed negative significant relationship with normal-FBS in total participant group and female group (Table-IV).

Chest-circumference showed a negative non-significant association with elevated-FBS across all groups. Similar relationship of chest size was noted with normal-FBS and reduced-FBS in the total participants and only females with normal-FBS. At the same time, all other study groups of normal-FBS and reduced-FBS expressed a non-significant positive association with chest size (Table-IV).

## DISCUSSION

The predominance of normal-weight participants aligns with global epidemiological trends. However, the underrepresentation of overweight/obese (9.55%) contrasts with the rising prevalence of obesity worldwide, particularly in developing countries.^12^ Gender disparities, with females predominating in the overweight/obese category in the present study, may reflect sociocultural, hormonal, and behavioral differences influencing weight gain. Estrogen’s role in adipose tissue distribution may partially explain this finding.^13^

Our findings indicated that females had a lower average BMI than males in the overweight and obese categories. This unexpected result may be due to methodological differences or sample characteristics, such as varying obesity classification thresholds and muscle-to-fat ratios. Additionally, BMI has limitations as a health metric since it does not differentiate between lean mass and fat mass.^14^ The higher prevalence of normal HR (50.31%) is encouraging, given the association of elevated HR with increased cardiovascular risk.^15^ Gender differences, with females exhibiting higher HRs in the normal range, are consistent with established physiological norms. Women typically have higher baseline HRs due to smaller heart size, higher stroke volume, and greater sympathetic activation.^16^

The prevalence of normal systolic BP (63.05%) reflects the study’s younger demographic, as younger people generally have lower blood pressure due to better vascular elasticity. Additionally, the lower systolic blood pressure in females aligns with previous research, indicating that hormonal factors, particularly estrogen’s vasodilatory effects, may play a role.^17^ The absence of significant gender differences in elevated systolic-BP categories remains inconsistent with literature as both genders in hypertension prevalence, awareness, and treatment outcomes.^18^,^19^

The high prevalence of normal FBS at 63.69% is reassuring, but the significant gender differences in elevated levels are concerning. Males tend to have higher FBS, which aligns with studies suggesting they are more susceptible to insulin resistance due to abdominal fat and hormonal factors. Furthermore, the inverse relationship between elevated and borderline heart rate categories reflects research on obesity-related autonomic dysfunction, showing decreased parasympathetic and increased sympathetic activity.^20^ Conversely, the positive association in the normal HR category may reflect compensatory mechanisms or measurement variability. The inverse relationships between FBS and BP challenge the conventional understanding that hyperglycemia is typically associated with hypertension due to endothelial dysfunction and oxidative stress.^21^ The Present study expressed an inverse relationship between BMI and HR in overweight/obese participants; similarly, a Chinese study claimed similar findings in overweight/obese Asian patients. The study also declared that such patients are more likely to have cardiovascular risk factors, especially coronary artery disease.^22^ The current results also expressed a direct association of BMI with BP in overweight or obese participants, quite similar to these findings. Another recent study concluded the same association in the South Asian population and also suggested that these individuals are more prone to hypertension.^23^

Related to the association of anthropometric measurements, i.e., arm and chest circumference, at present, a significant direct relationship is found in overweight or obese participants, in the same lines a similar study conducted with Palestinian adults expressed a similar association, however, in that study the used anthropometric measurements were waist and hip circumference, and total body fat.^24^ Quite relatable to our findings, that study also claimed a direct association of BP in such individuals. Moreover, these individuals are also more likely to be hypertensive.^24^ A similar study but with a lesser age group also expressed a positive link between arm circumference and overweight or obese children.^25^

Our findings revealed significant differences in study parameters across various BMI categories. The study emphasizes the importance of gender-specific anthropometric factors in assessing cardiovascular and metabolic health risks. Overweight or obese females generally have a lower BMI but higher FBS compared to males. We found positive correlations between FBS and heart rate, particularly in normal and borderline FBS categories. This highlights the need for gender-specific analyses to enhance health assessments and develop targeted intervention strategies.

### Limitations:

The present study did not assess the role of key variables like diet, physical activity, medication use, and psychological stress. Moreover, the participants’ demographic homogeneity may restrict the generalizability of the findings to more diverse populations.

## CONCLUSION

Females were more overweight/obese in number but with BMI 14.9% lower than males. Males had higher HR and BP values, while elevated FBS was more common in females. HR positively correlated with BP and chest-circumference in males and FBS in both genders but negatively associated with arm-circumference in males. Systolic-BP had a strong association with HR in females. High BMI was linked to larger arm/chest-circumferences and elevated BP, particularly in females. FBS strongly correlates with BMI in females, while BP and HR in males. In overweight or obese subjects, positive association of arm or chest circumference was found with BMI, and such subjects expressed a significant positive association with BP and negative association with HR.

### Authors’ Contribution:

**AS:** Conceived, designed and did statistical analysis & editing of manuscript, is responsible for integrity of research.

**AN and AR:** Did data collection and manuscript writing. Critical analysis

**AS, AN and AR:** Critical review and final approval of manuscript.
